# Fecal microbiota transplantation reverses antibiotic and chemotherapy-induced gut dysbiosis in mice

**DOI:** 10.1038/s41598-018-24342-x

**Published:** 2018-04-18

**Authors:** Quentin Le Bastard, Tonya Ward, Dimitri Sidiropoulos, Benjamin M. Hillmann, Chan Lan Chun, Michael J. Sadowsky, Dan Knights, Emmanuel Montassier

**Affiliations:** 1 Université de Nantes, Microbiotas Hosts Antibiotics and bacterial Resistances (MiHAR), Nantes, 44000 France; 2 Biotechnology Institute, University of Minnesota, Saint Paul, Minnesota 55108 USA; 3 Department of Computer Science and Engineering, University of Minnesota, Minneapolis, Minnesota 55455 USA; 4 Department of Civil Engineering and National Resources Research Institute, University of Minnesota Duluth, Duluth, Minnesota USA; 5 Department of Soil Water & Climate, and Department of Plant and Microbial Biology, University of Minnesota, Saint Paul, Minnesota 55108 USA

## Abstract

Fecal microbiota transplantation (FMT) is now widely used to treat recurrent *Clostridium difficile* infection, but has been less studied as a means to restore microbiome diversity and composition following antibiotic or chemotherapy treatments. The purpose of our study was to assess the efficacy of FMT to reverse antibiotic- and chemotherapy-induced gut dysbiosis in a mouse model. C57BL/6J mice were treated with ampicillin for 1 week and/or received a single intraperitoneal injection of 5-Fluorouracil. Fresh stool was collected and analyzed using shotgun metagenomics and the Illumina sequencing platform. Ampicillin caused a significant and immediate decrease in bacterial species richness and diversity that persisted for one week. In mice that received FMT, disruption of the intestinal microbiota was reversed immediately. Antibiotic and chemotherapy administration caused significant alteration in species distribution, including a decrease in the relative proportions of *Clostridium scindens* and *Faecalibacterium prausnitzii*, and an increase in known pathogenic species. In mice receiving FMT, we observed a significant increase in species known to exhibit anti-inflammatory properties. Moreover, chemotherapy led to a critical decrease in key ‘health-promoting’ species and to an altered functional profile, especially when chemotherapy was administered in tandem with antibiotics, and that FMT can ameliorate these effects.

## Introduction

Cancer patients, especially those with hematological malignancies, receive high doses of chemotherapeutic agents that often cause for gastrointestinal mucositis^1^. This side effect can enable bacterial translocation leading to bloodstream infection (BSI), a major cause of morbidity and mortality in cancer patients^2^. In previous studies, the incidence of BSI was reported to be 20% to 60% in patients receiving high dose chemotherapy, and sepsis-associated mortality ranged from 9% to 31%^3–5^.

It was previously reported that the intestinal microbiota are drastically altered following chemotherapy, and that intestinal microbiota composition in pretreatment individuals can be used to predict the onset of subsequent bacteremia^6,7^. These authors reported that several taxa, including *Barnesiellaceae*, *Christensenella* and *Faecalibacterium*, were less abundant in patients who developed a subsequent BSI. It was postulated that these strains could play a protective role against BSI.

Myelosuppresive chemotherapy and some malignancies themselves lead to immunosuppression and to increased susceptibility to bacterial infections. Thus, prophylactic and empirical antibiotics are commonly used to reduce morbidity and mortality^4,8^. Antibiotic treatments also adversely affect the gut microbiota and enable colonization with multidrug-resistant bacteria^9^.

Fecal microbiota transplantation (FMT) is now widely used to treat recurrent *Clostridium difficile* infection (rCDI) with clinical success rates >90%^10^. Intestinal microbiota composition following FMT is restored to a healthy state similar to the donor^11,12^. A mouse model demonstrated that FMT in vancomycin-resistant *Enterococcus faecium* (VRE)-colonized mice eliminates infection by pathogenic microorganism, and that elimination of this colonization was correlated with restoration of a fecal flora that contains *Barnesiella*^13^.

Based on findings reported above, we hypothesized that a diverse and healthy microbial ecosystem plays a role in maintaining the gut barrier that prevents translocation of pathogenic microorganisms, and that administering FMT to patients with existing dysbiosis, induced by antibiotic or chemotherapy, may reduce the risk of this complication. Therefore, the purpose of our study was to: (1) assess the efficacy of FMT in a mouse model to reverse antibiotic- and chemotherapy-induced gut dysbiosis, and (2) define bacterial strains that may be associated with the restoration of a healthy state. To achieve our goal, we transplanted fecal microbiota from untreated mice into mice whose endogenous microbiota had been disrupted by chemotherapy alone, or with antibiotics plus chemotherapy, and compared the intestinal microbiota restoration with mice that did not received FMT.

## Results

### Untreated mice

In mice that did not receive any treatment (n = 5), fecal samples collected longitudinally had comparable alpha diversity metrics for Chao1 (ANOVA, p = 0.95) and numbers of observed unique functions (ANOVA, p = 0.8) [Supplementary Fig. S1A]. Principal coordinates analysis (PCoA) of Bray Curtis dissimilarities, did not show significant differences in fecal microbiota architecture between the different time points (p = 0.41, Supplementary Fig. S1B).

### Richness and diversity were restored by FMT

**Figure 1:**
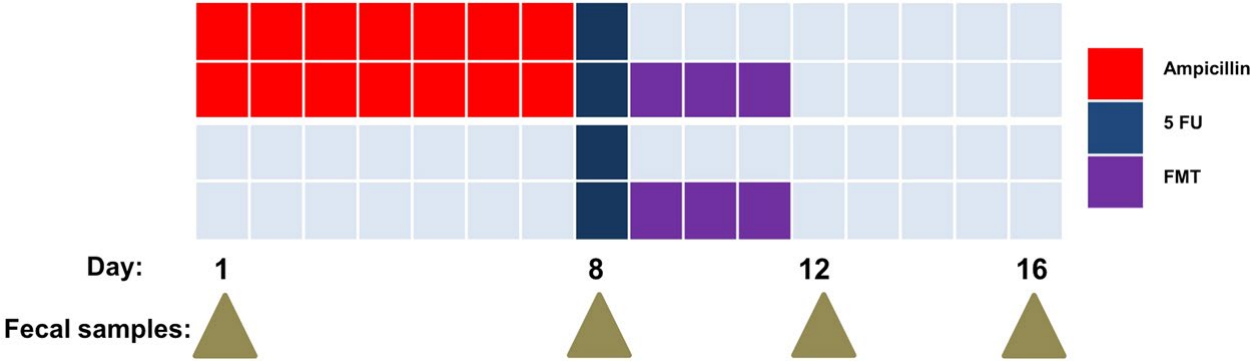
Experimental design of the study. The first group of mice (n = 4) received Ampicillin from days 1 to 7 and chemotherapy on day 8. The second group (n = 4) received the same regimen and FMT from days 9 to 11. The third group of mice (n = 5) received chemotherapy on day 8 and the last group of mice (n = 5) received chemotherapy on day 8 and FMT from days 9 to 11. A control group received no treatment. Fecal samples were collected on day 1, 8, 12 and 16.

**Figure 2:**
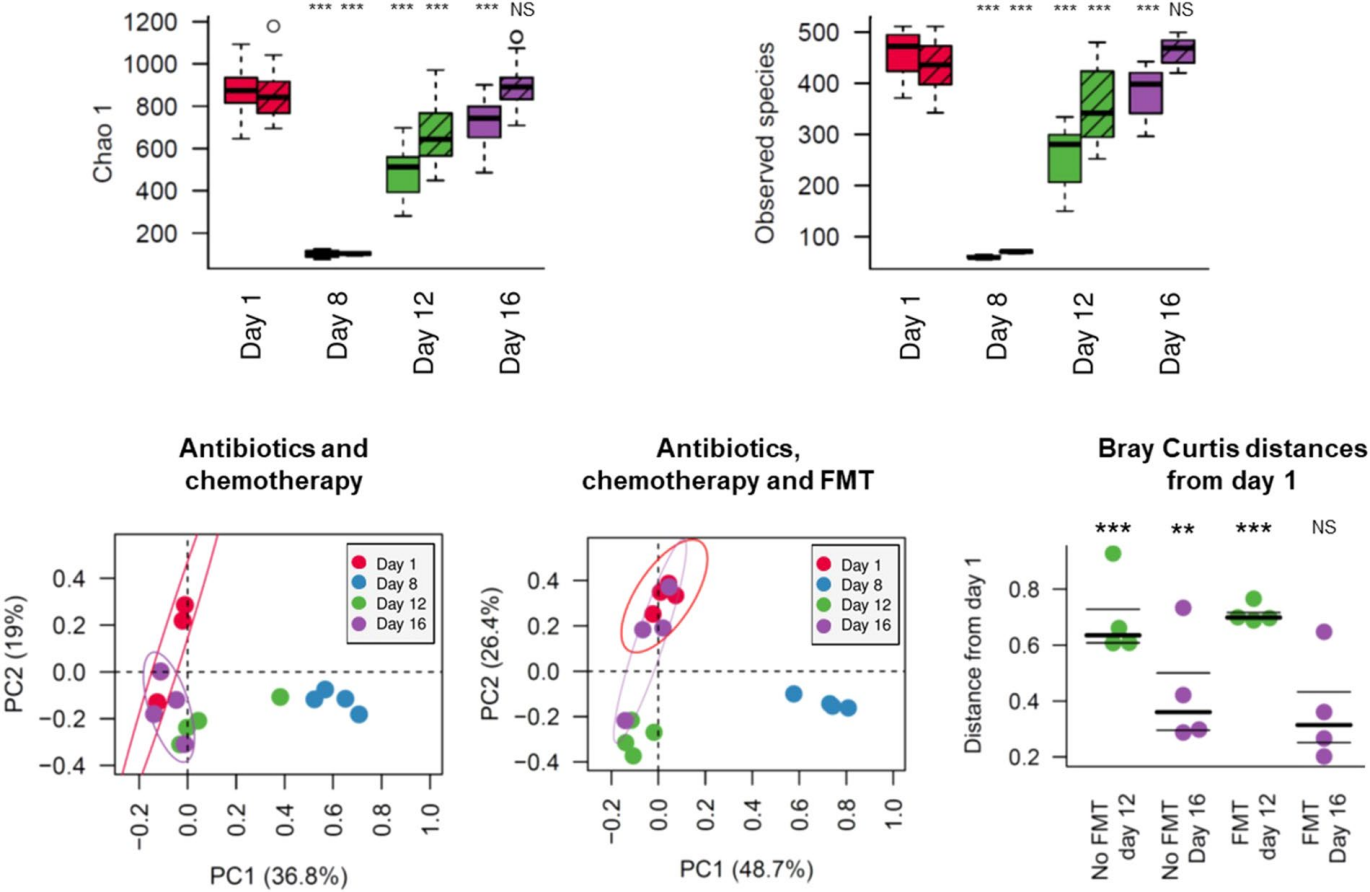
Species alpha and beta diversity disruption induced by antibiotic and chemotherapy are corrected by FMT. (**A**) Alpha diversity indexes are represented at different collection time points in mice exposed to antibiotics from days 1 to 7 and chemotherapy on day 8 (colored) and mice that received the same regimen plus FMT from days 9 to 11 (striped). For each day, indexes are compared to those on day 1. Alpha-diversity decreases after antibiotics and chemotherapy administration and was still significantly decreased one week after treatment discontinuation in mice that did not receive FMT. In contrast, mice receiving FMT had gut microbial diversity that was no longer significantly different from the pretreatment state. (**B**) Species beta-diversity comparison of the gut microbiota as represented by principal coordinate analysis (PCoA) of Bray Curtis distances for non-FMT mice (left) and FMT-treated mice (right). Each point represents a single mouse sample and day of collection is denoted by its color. Colored circles represent the 95% confidence interval. In non-FMT mice, samples collected on day 16 are dispersed around day 1 samples while in FMT mice, day 16 samples are grouped around day 1 samples, suggesting Bray Curtis distances from days 1 to day 16 samples are lower in FMT mice. Proportion of variance explained by each principal coordinate axis is denoted in the corresponding axis label. (**C**) Beta diversity Bray Curtis distances variations from day 1 samples. Each point represents the distance from day 1 corresponding sample. Mice were exposed to antibiotics and chemotherapy with fecal transplantation (Abt-Chem-FMT day 12, Abt-Chem-FMT day 16) or without (Abt-Chem day 12, Abt-Chem day 16). Distances between day 12 or 16 samples and day 1 samples appear significant in non FMT mice while they are non-significant in FMT exposed mice, suggesting FMT restored beta diversity. Bray Curtis distances are compared for day 12 and 16 to control group samples using ANOVA test. The symbols: ***Denote *p* < 0.001, **p < 0.01 and *p < 0.05, compared for day 12 and 16 to control group samples using ANOVA test.

To investigate the influence of antibiotic and/or chemotherapy administration, eight mice were treated with ampicillin during week 1, and one intraperitoneal injection of 5-FU on the eighth day. Four mice in this group (identified as “Abt-Chem-FMT group”, n = 4) received FMT from an untreated donor mouse for three 3 days, starting one day after the chemotherapy administration. The four remaining mice (identified as “Abt-Chem group”, n = 4) received oral gavage of water during the same 3 days period (Fig. 1). Ampicillin given from days 1 to 7 caused a significant and immediate decrease in microbial species richness and diversity, as measured by alpha diversity metrics (Day 1 Abt-Chem and Abt-Chem-FMT groups vs Day 8 Abt-Chem group, Chao 1 and Unique observed species, *p* < 0.001 both). After chemotherapy administration on day 8, alpha diversity was still reduced relative to control mice (Day 1 Abt-Chem and Abt-Chem-FMT groups vs Day 12 Abt-Chem group, Chao 1 and Unique observed species, *p* < 0.001 both). Moreover, by one week after antibiotic and chemotherapy discontinuation, mice that did not receive FMT did not recover microbial richness and diversity seen in pretreatment mice (Day 1 Abt-Chem and Abt-Chem-FMT groups vs Day 16 Abt-Chem group, Chao 1 and Unique observed species, *p* < 0.001 both, Fig. 2A). PCoA of Bray-Curtis dissimilarities showed a profound disruption of the architecture of the intestinal microbiota after antibiotic treatment (ANOSIM Day 1 Abt-Chem and Abt-Chem-FMT groups vs Day 8 Abt-Chem group, *p* = 0.001), which persisted after chemotherapy administration (ANOSIM Day 1 Abt-Chem and Abt-Chem-FMT groups vs Day 12 Abt-Chem group, *p* = 0.004) and 1 week after discontinuation of antibiotic and chemotherapy treatment in mice that did not receive FMT (ANOSIM Day 1 Abt-Chem and Abt-Chem-FMT groups vs Day 16 Abt-Chem group, *p* = 0.007, Fig. 2B).

In mice that received FMT after antibiotic and chemotherapy treatment, fecal samples collected before antibiotic and chemotherapy treatment and one week after antibiotic and chemotherapy discontinuation did not have significant differences in terms of diversity, as indicated by alpha diversity metrics for Chao1 (Day 1 Abt-Chem and Abt-Chem-FMT groups vs Day 16 Abt-Chem-FMT group, Chao 1 *p* = 0.08, Unique observed species, *p* = 0.55, Fig. 2A). A PCoA of Bray-Curtis dissimilarities showed that the disruption of the intestinal microbiota was significantly different immediately after FMT (ANOSIM Day 1 Abt-Chem and Abt-Chem-FMT groups vs Day 12 Abt-Chem-FMT group, *p* = 0.001) but no longer significant one week after antibiotic and chemotherapy discontinuation (ANOSIM Day 1 Abt-Chem and Abt-Chem-FMT groups vs Day 16 Abt-Chem-FMT group, *p* = 0.43, Fig. 2B) PCoA did not show significant differences in terms of diversity between untreated mice and post FMT samples (ANOSIM: untreated mice vs Day 16 Abt-Chem-FMT group, *p* = 0.076). Comparison of beta diversity among samples collected on day 1 showed that days 12 and 16 samples from mice that did not receive FMT were significantly different from each other (ANOSIM Day 1 Abt-Chem and Abt-Chem-FMT groups vs Day 12 Abt-Chem group and Day 16 Abt-Chem group: *p* = 0.01 and *p* = 0.02, respectively), they were also significantly different on day 12 (*p* = 0.003) in mice that received FMT whereas samples collected on 16 from mice that received FMT did not differ significantly (*p* = 0.43, respectively, Fig. 2C), indicating that FMT enabled recovery of gut microbial diversity.

### Taxonomic balance was restored by FMT

**Figure 3:**
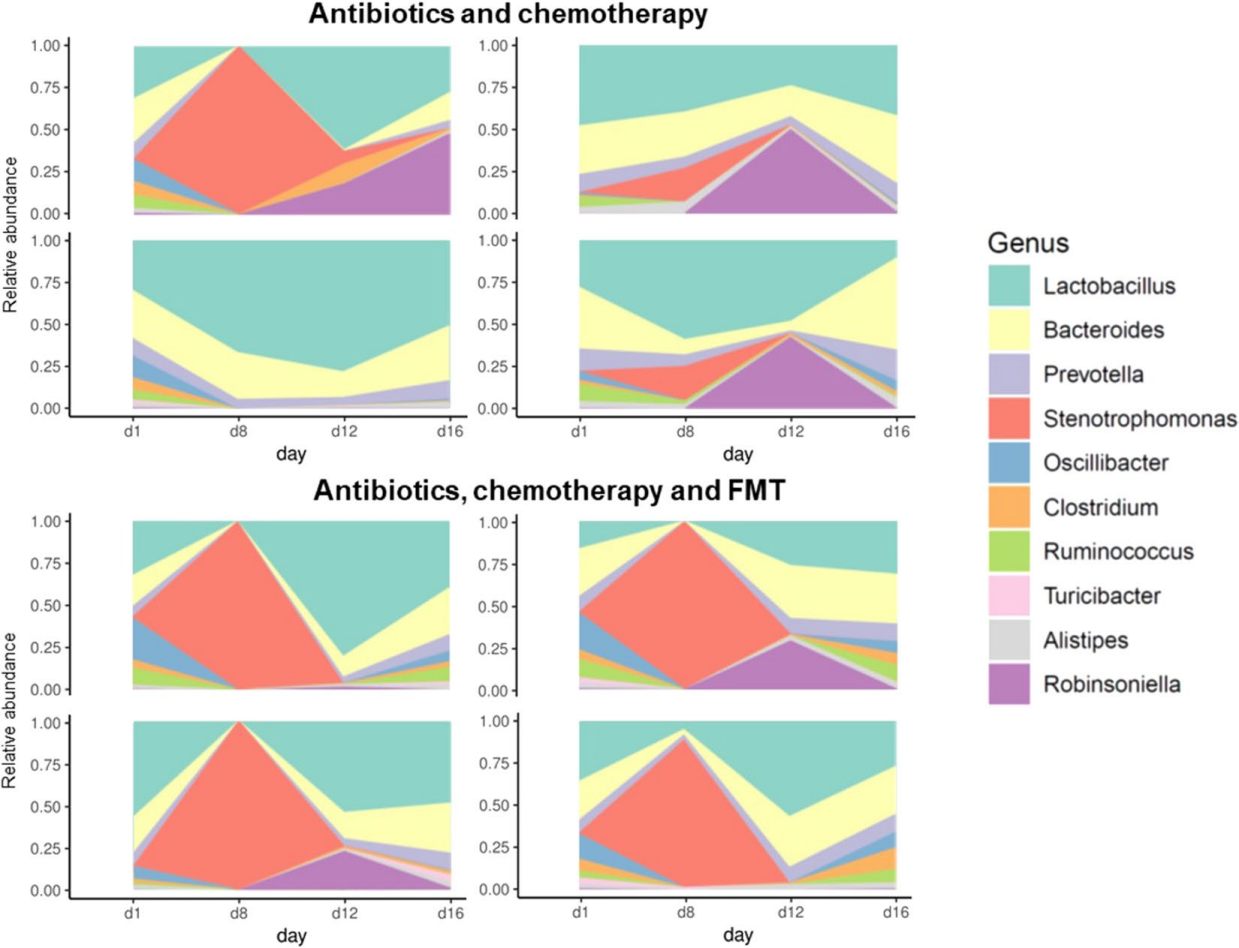
Distribution of bacterial genera is strongly disrupted by antibiotics and chemotherapy and restored by FMT. Each plot shows, for one mouse, the longitudinal follow up of the 10 most abundant bacterial genera. The four plots on top represent mice exposed to antibiotic treatment from days 1 to 7 and chemotherapy on day 8. The four bottom plots represent mice that received the same regimen and FMT from days 9 to 11.

Following antibiotic treatment alone, we found a large change in microbiota at the genus level, with a significant increases of 5 genera including *Xanthomonas* and *Stenotrophomonas*, and a significant decrease of several genera, including *Lactobacillus*, *Bacteroides*, *Prevotella*, *Barnesiella*, *Butyricimonas*, *Eubacterium*, *Lachnospira*, *Bifidobacterium*, *Faecalibacterium*, *Ruminococcus* and *Blautia* (Untreated mice vs Day 8 Abt-Chem and Abt-Chem-FMT groups, Mann-Whitney U test FDR corrected *p* < 0.05, Fig. 3, Supplementary Table S1). Following antibiotic treatment alone, there was a significant increase of 22 species including *Stenotrophomonas maltophilia*, *Acinetobacter baumannii*, *Enterococcus faecium*, *Chlamydia trachomatis and Clostridium difficile* and significant decrease of 1304 species, including several *Roseburia* species (*R. faecis*, *R. intestinalis*, *R. hominis*), several *Eubacterium* species (*E. halii*, *E. plexicaudatum*, *E. ventriosum*, *E. rectale*), *Butyricicoccus pullicaecorum*, *Ruminococcus bromii*, *Lactobacillus johnsonii*, *Barnesiella viscericola*, *Faecalibacterium prausnitzii*, *Clostridium butyricum* and *Clostridium scindens* (Untreated mice vs Day 8 Abt-Chem and Abt-Chem-FMT groups, Mann-Whitney U test FDR corrected *p* < 0.05) [Supplementary Table S2].

Antibiotic plus chemotherapy administration resulted in significant changes at the species level, with a decrease of numerous species, including *Faecalibacterium prausnitzii*, *Eubacterium plexicaudatum*, *Eubacterium rectale*, *Butyricicoccus pullicaecorum*, *Roseburia faecis and Roseburia intestinalis* and an increase of 16 species, including *Enterococcus faecalis*, *Stenotrophomonas maltophilia*, *Enterococcus gallinarum and Staphylococcus aureus*. *Clostridium scindens* abundance was not significantly modified after chemotherapy. (Untreated mice vs Day 12 Abt-Chem group, Mann-Whitney U test FDR corrected *p* < 0.05) [Supplementary Table S3]. Furthermore, one week after discontinuation of antibiotics and chemotherapy taxonomic profile at genus level with a significant decrease of *Ruminococcus*, *Eubacterium* and an increase of *Escherichia*, *Shigella* and *Citrobacter* genera. Taxonomic profile at the species level still remained disrupted relative to controls, with a significant decrease of five species including *Butyricicoccus pullicaecorum* and *Eubacterium plexicaudatum*. (Untreated mice vs Day 16 Abt-Chem goup, FDR corrected p value < 0.05) [Supplementary Table S4], relative to the untreated control. Also, we observed a significant increase of *Escherichia coli* and *Streptococcus agalactiae* and a decrease in *Roseburia faecis*, several *Eubacterium* species and *Ruminococcus* species. (Untreated mice vs Day 16 Abt-Chem goup, FDR corrected p value < 0.20).

Mice receiving FMT after antibiotic and chemotherapy administration had significant and immediate changes at species level, with the decrease of 176 species (untreated mice vs Day 12 Abt-Chem-FMT group, Mann-Whitney U test FDR corrected *p* < 0.05). Immediately after FMT, *Faecalibacterium prausnitzii* levels were no longer significantly different when compared to untreated mice (untreated mice vs Day 12 Abt-Chem-FMT group, Mann-Whitney U test FDR corrected *p* = 0.07) [Supplementary Table S5]. One week after the discontinuation of antibiotics and chemotherapy, gut bacterial taxonomic profiles, at species level, was no longer different when compared to untreated mice profile (Untreated mice vs Day 16 Abt-Chem-FMT group, Mann-Whitney U test FDR corrected *p* < 0.05), indicating that FMT allowed mice to recover specific depleted taxa as well as the overall pre-treatment microbiome profile. We did not observe any trend at a lower level of sensibility (Untreated mice vs Day 16 Abt-Chem-FMT group, Mann-Whitney U test FDR corrected *p* < 0.20),

Furthermore, between day 8 and day 16, we observed a significant increase in *Escherichia coli* in mice that did not receive FMT whereas we observed a significant decrease in mice that received FMT (Day 8 Abt-Chem group vs Day 16 Abt-Chem group, Mann-Whitney U test FDR corrected *p* < 0.05).

**Figure 4:**
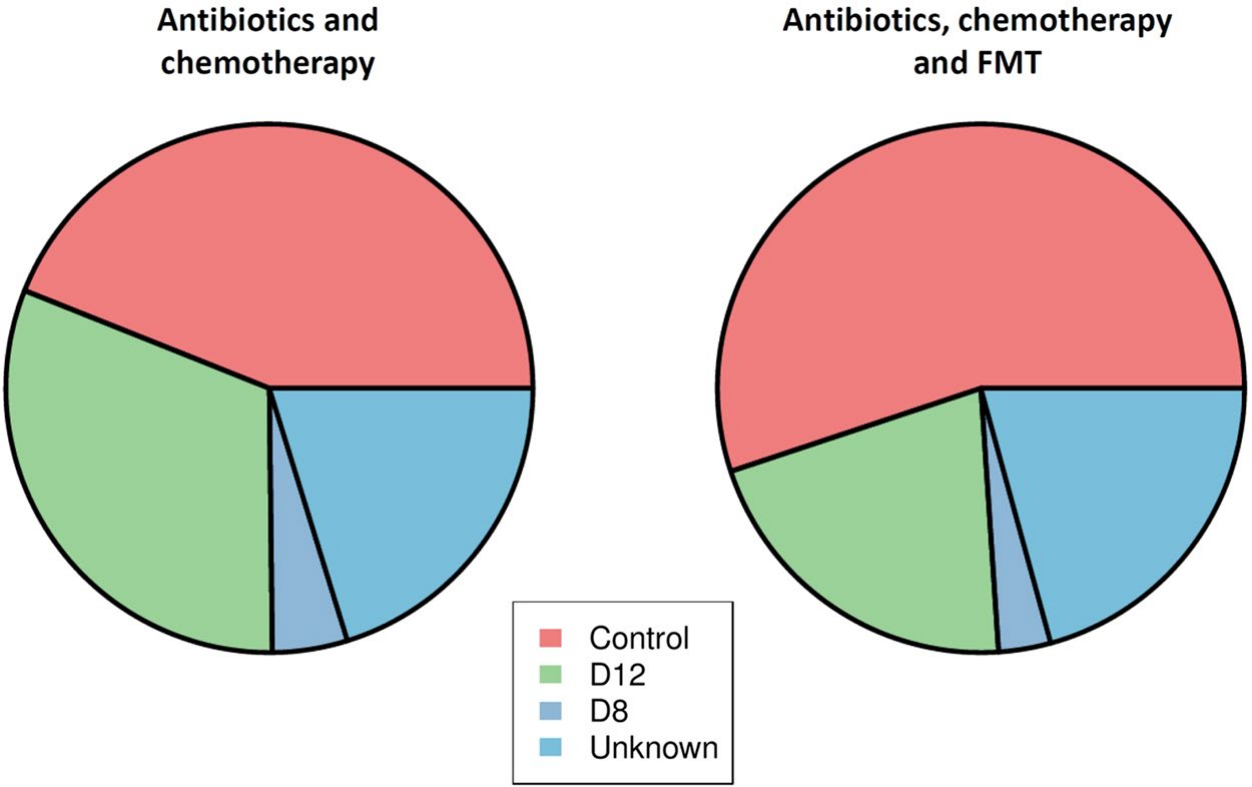
Intestinal microbiota from untreated mice represent the primary contribution (55%) to mice that received FMT and is higher than in mice that did not received FMT (44%). SourceTracker was used to estimate the proportion of several source communities (control mice in red, day 8 samples in dark blue, day 12 in green and unknown source in light blue) that contributes in the constitution of samples collected one week after antibiotic and chemotherapy from mice that did not received FMT (left) and mice that received FMT (right).

Microbial source-tracking was done using the Bayesian SourceTracker pipeline on samples collected one week after antibiotic and chemotherapy discontinuation in mice that did or did not receive FMT^14^. SourceTracker is a probabilistic algorithm that predicts the contribution of several microbial source communities (samples collected in untreated mice and samples collected on day 8 and 12) in a set of samples (samples collected on day 16). The intestinal microbiota from untreated mice represented the primary contribution (55% average, *p* < 0.05) in mice that received FMT, the contribution of the intestinal microbiota from untreated mice, in mice that did not receive FMT, was lower (44% average, *p* < 0.05). Intestinal microbiota from post antibiotic treatment samples was higher (5% average from Day 8 and 31% average from Day 12) in mice that did not received FMT than in mice that received FMT (3% average from Day 8 and 21% average from Day 12) (Fig. 4).

### Functional dysbiosis was corrected by FMT

**Figure 5:**
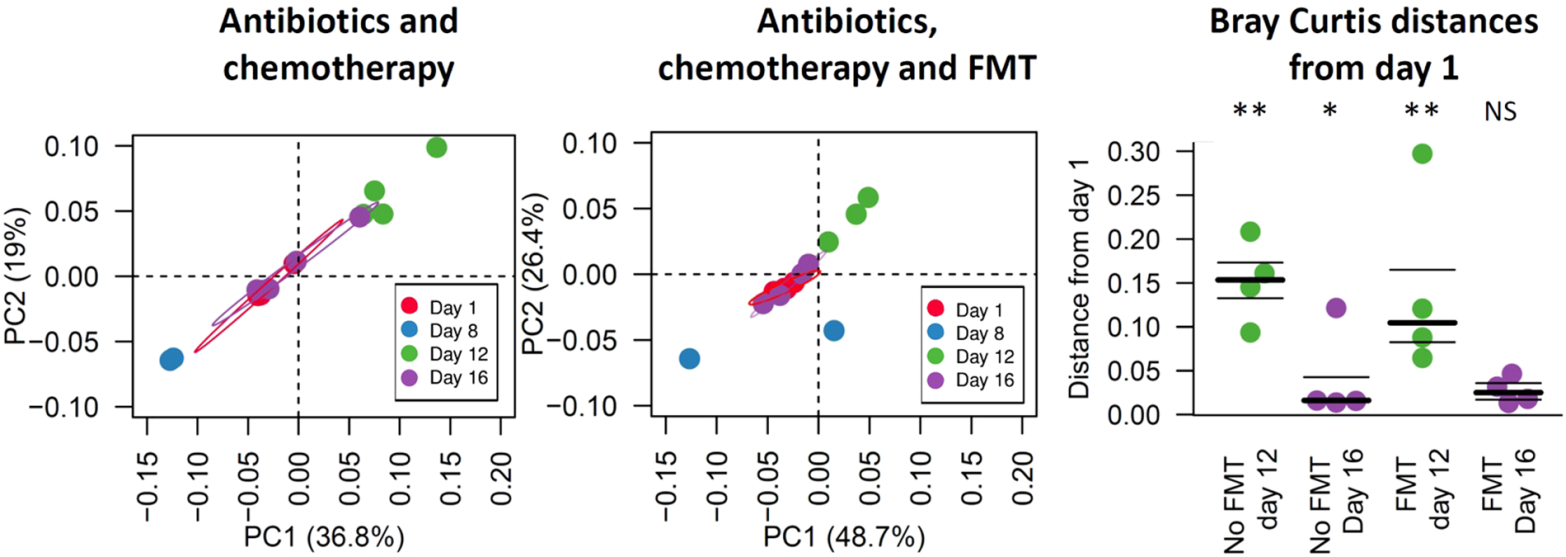
Disruption of functional beta diversity induced by antibiotics and chemotherapy is corrected by FMT. (**A**) Beta diversity of the functional repertoire (KEGG) comparison of the gut microbiomes of fecal samples collected in mice exposed to antibiotic treatment from days 1 to 7, chemotherapy on day 8 (left) and mice that received FMT from days 9 to 12 (right). Principal coordinate analysis (PCoA) of Bray Curtis distances. Each point represents a sample from one mouse and the day of collection is denoted by its color. Colored circles contain 50% of the corresponding day samples. In non-FMT mice, samples collected on day 16 are dispersed around day 1 samples while in FMT mice, day 16 samples are grouped around day 1 samples, suggesting Bray Curtis distances from day 1 to day 16 samples are lower in FMT mice. Proportion of variance explained by each principal coordinate axis is denoted in the corresponding axis label. One sample collected on day 12 in FMT group (mouse 228 exposed to antibiotic treatment, chemotherapy and FMT) appears as an outlier. (**B**) Beta diversity Bray Curtis distances of the functional repertoire (KEGG) from day 1 samples of mice exposed to antibiotics and chemotherapy with (FMT day 12, FMT day 16) or without (No FMT day 12, No FMT day 16) fecal transplantation. Each point represents distance from day 1 sample for each mouse. Bray Curtis distances are compared for day 12 and 16 to control group samples using ANOVA test. Symbol: *Denotes *p* < *0.05*.

Beta-diversity plots generated from Bray Curtis distance metric of predicted KEGG Orthologs showed a profound disruption of the functional architecture of the intestinal microbiota after antibiotic treatment (Day 1 vs Day 8 Abt-Chem and Abt-Chem-FMT groups, *p* = 0.001). These changes persisted after chemotherapy administration (Day 1 Abt-Chem and Abt-Chem-FMT groups vs Day 12 Abt-Chem group, *p* = 0.003) and one week after antibiotic and chemotherapy discontinuation in mice that did not receive FMT (Day 1 Abt-Chem and Abt-Chem-FMT groups vs Day 16 Abt-Chem group, *p* = 0.035, Fig. 5A).

Following antibiotic treatment, we identified numerous putative KEGG orthologs that showed a significant difference in abundance when compared to untreated mice (Untreated mice vs Day 8 Abt-Chem and Abt-Chem-FMT groups, Mann-Whitney U test FDR-corrected *p* < 0.05). Of these, 96.4% were decreased after antibiotic treatment. These KEGG orthologs were predominantly associated with metabolism and environmental information processing. More specifically, they were mainly involved in carbohydrate and lipid metabolism, energy metabolism, nucleotide and amino acid metabolism, environmental information processing, and genetic information processing. Antibiotic treatment increased 20 orthologs, mainly those involved in nucleotide and amino acid metabolism (40%) [Supplementary Table S6]. Following chemotherapy administration, the functional dysbiosis persisted and 126 KEGG orthologs increased (Untreated mice vs Day 12 Abt-Chem group, Mann-Whitney U test FDR-corrected *p* < 0.05) [Supplementary Table S7]. Furthermore, one week after antibiotics and chemotherapy discontinuation, we did not identify any significant differences in abundance when compared to untreated mice (Untreated mice vs Day 16 Abt-Chem group, Mann-Whitney U test FDR-corrected *p* < 0.05). [Supplementary Table S8].

To further characterize these functional differences, we performed statistical analyses on tables annotated using Enzyme Commission (EC) codes. Immediately after antibiotic administration, 388 and 8 EC decreased and increased, respectively. Decreased EC enzymes involved those involved in butyrate and pyruvate metabolism: 6.2.1.2: Butyrate CoA ligase, 2.7.1.40: Pyruvate kinase, 2.8.3.8: Acetate CoA-transferase (Untreated mice vs D8 Abt-Chem and Abt-Chem-FMT groups, Mann-Whitney U test FDR-corrected *p* < 0.05) [Supplementary Table S9]. Following chemotherapy administration, alterations were persistent with 11 EC decreased and 126 increased (Untreated mice vs Day 12 Abt-Chem group, Mann-Whitney U test FDR-corrected *p* < 0.05) [Supplementary Table S10]. One week after antibiotics and chemotherapy discontinuation we did not identify any significant differences in abundance when compared to untreated mice (Untreated mice vs Day 16 Abt-Chem group, Mann-Whitney U test FDR-corrected *p* < 0.05) [Supplementary Table S11].

Beta-diversity plots generated from Bray Curtis dissimilarities of KEGG orthologs tables in mice that received FMT showed that, diversity was no longer significantly different from pretreatment state one week after antibiotics and chemotherapy discontinuation. (ANOSIM Day 1 Abt-Chem and Abt-Chem-FMT groups vs Day 16 Abt-Chem-FMT group, *p* = 0.16) (Fig. 5A). Furthermore, when comparing beta diversity from samples collected on day 1, we found that day 12 and day 16 samples from mice that did not receive FMT were significantly different (*p* = 0.002 and *p* = 0.001. respectively), whereas samples collected from mice that received FMT were significantly different on day 12 but not on day 16 were (*p = *0.001 and *p* = 0.52 respectively, Fig. 5B). Moreover, we did not identify neither differentiated KEGG orthologs nor EC among samples collected from untreated mice and post FMT samples (untreated mice vs Day 16 Abt-Chem-FMT group, FDR-corrected *p* > 0.05 both).

### Chemotherapy alone caused less, but significant, disruption

To investigate the influence of chemotherapy alone, we subjected another group of 10 mice to a single intraperitoneal injection of 5-Fluorouracil. Five of these mice (identified as “Chem-FMT group”, n = 5) subsequently received FMT for 3 days, starting one day after the chemotherapy administration and 5 mice (identified as “Chem group”, n = 5) received oral gavages of water during the same 3 days period. A PCoA of species level abundances, done using Bray-Curtis distance metric, showed a disruption of the architecture of the intestinal microbiota. This dysbiosis persisted one week after chemotherapy administration, although the differences were less significant than those observed in the group receiving both antibiotics and chemotherapy (Day 1 Chem and Chem-FMT groups vs Day 9, *p* = 0.001, Supplementary Fig. S2). Following chemotherapy administration, we did not observe any significant modification of the taxonomic profiles at genus level (Day 9 Chem group vs untreated mice, Mann-Whitney U test FDR corrected, all *p* > 0.20). At species level we found a significant modification of 72 species abundance including a decrease in *Eubacterium ventriosum*, *Ruminococcus sp*. We also observed an increase of *Barnesiella viscericola* and *Lactobacillus johnsonii* (Day 9 Chem group vs untreated mice, Mann-Whitney U test FDR corrected *p* < 0.20). One week after chemotherapy discontinuation, taxonomic profile was still modified with 5 species increased (Day 16 Chem group vs untreated mice, Mann-Whitney U test FDR corrected *p* < 0.05). After chemotherapy treatment, we identified 109 EC that exhibited a significant difference in abundance when compared to untreated mice (Untreated mice vs Day 9 Chem group, Mann-Whitney U test FDR corrected *p* < 0.20). [Supplementary Table S12, Supplementary Table S13].

In mice that received FMT after chemotherapy (from Days 2 to 4), PCoA of species level abundances, done using the Bray-Curtis distance metric showed that the disruption of the intestinal microbiota was no longer significant one week after FMT (Day 1 Chem and Chem-FMT groups vs Day 9 Chem-FMT group, *p* = 0.435). Comparison of taxonomic (at genus and species levels) and functional profiles (using KEGG modules and EC), did not show differentiated features between fecal samples collected pretreatment and one week after FMT (Untreated mice vs Day 9 and Day 16 Chem-FMT group, Mann-Whitney U test FDR corrected *p* > 0.05).

## Discussion

In this study, we investigated the influence of FMT on fecal microbiota composition and structure in mice receiving antibiotics, chemotherapy, or both. We demonstrated that antibiotic treatment and chemotherapy administration together led to a profound taxonomic and functional imbalance that could be reversed within a week by fecal microbiota transplantation. We also described a significant, although less severe, taxonomic and functional dysbiosis in mice treated with chemotherapy alone. In our work, taxonomic profiles showed a dominance of Lactobacillus genera. Although Lactobacilli dominance in mouse microbiota is atypical, it has been previously described, with up to 80% of bacterial communities^13,15–17^. The same profiles were observed using both MetaPhlAn2^18^ and CLARK^19,20^ suggesting that such observed taxonomic profiles are not artifacts (Fig. S3).

**Figure 6:**
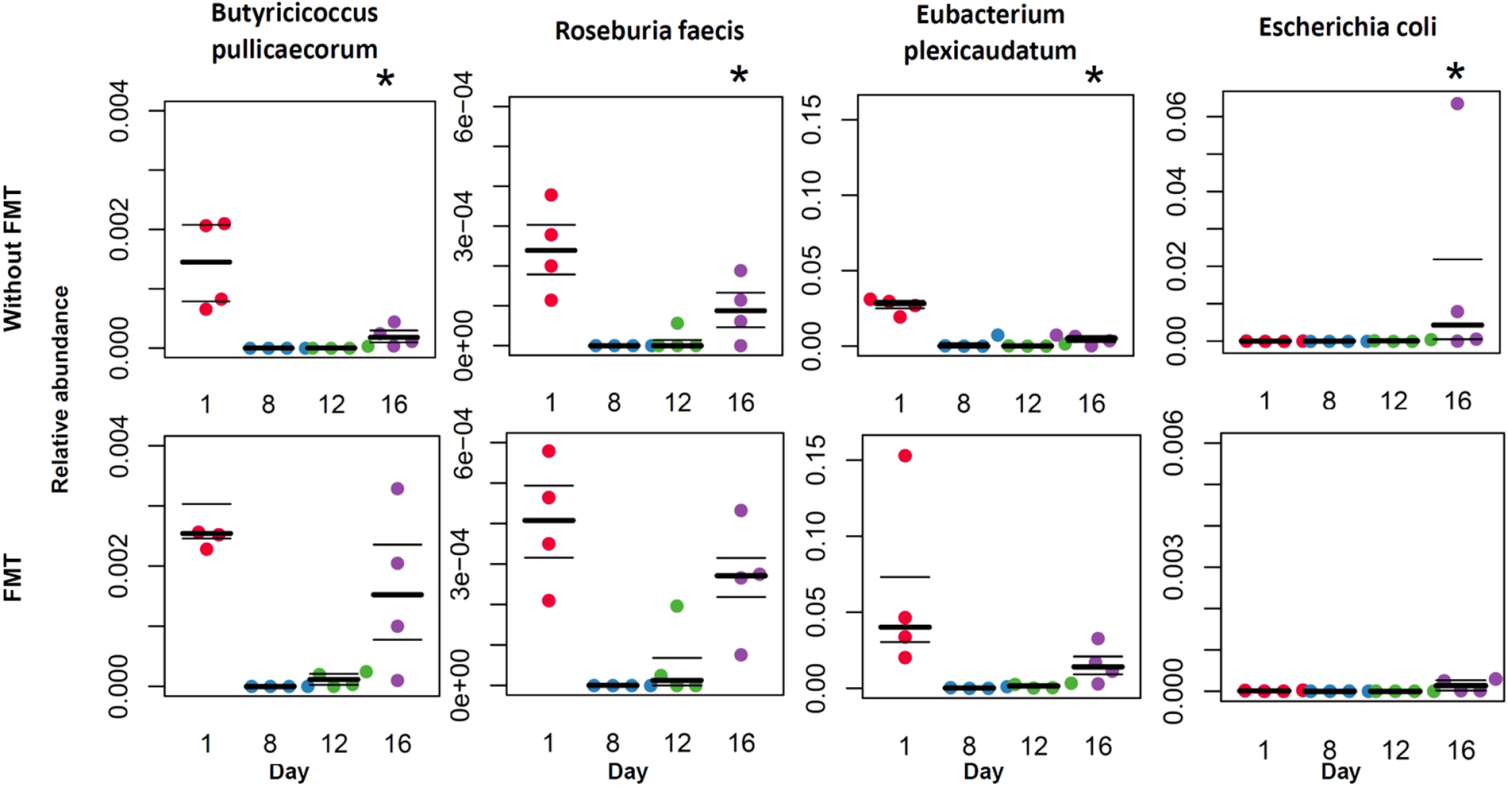
Relative abundance of species is modified after antibiotic and chemotherapy treatment and this disruption is corrected by FMT. Longitudinal follow up of selected “beneficial” species (*Butyricoccus pullicaecorum*, *Roseburia faecis* and *Eubacterium plexicaudatum*) and “pathogenic” species (*Escherichia coli*) in mice exposed to antibiotic treatment from days 1 to 7, chemotherapy on Day 8 (upper row) and mice that received FMT from day 9 to day 12 (lower row). Species relative abundance on day 16 are compared to control samples using FDR corrected p value. Symbols: *Denotes FDR corrected p value < *0.05*.

A longitudinal study showed that mice first experienced a decrease in presumed health-associated species, including several *Roseburia* species (*R. faecis*, *R. intestinalis*, *R. hominis*), several *Eubacterium* species (*E. halii*, *E. plexicaudatum*, *E. ventriosum*, *E. rectale*), *Butyricicoccus pullicaecorum*, *Ruminococcus bromii*, *Lactobacillus johnsonii*, *Barnesiella viscericola*, *Faecalibacterium prausnitzii*, *Clostridium butyricum* and *Clostridium scindens*. Secondly, there was an overgrowth of known potentially pathogenic bacteria (*Cytrobacter*, *Acinetobacter baumannii*, *Enterococcus faecium*, *Enterococcus faecalis*, *Staphylococcus aureus*, *Chlamydia trachomatis*, *Escherichia* genera (especially *Escherichia coli*), *and Clostridium difficile*, Fig. 6). Importantly, these potentially pathogenic organisms are among the most frequently isolated pathogens in blood culture of cancer patients^2^. *Shigella* species were also reported as increased in the microbiota of children with multiple sclerosis^21^. In mice that received FMT after antibiotic and chemotherapy treatment, intestinal microbiota dysbiosis was rapidly corrected and pathogenic bacteria did not significantly emerge in the gut. Thus, FMT restored a healthy and diverse intestinal microbiota that could potentially prevent the emergence of pathogenic bacteria and bacterial translocation from the gastrointestinal tract to the bloodstream^22^.

In mice receiving antibiotic and chemotherapy, we observed a depletion of organisms previously thought to confer anti-inflammatory benefits, such as *Faecalibacterium prausnitzii* and *Lactobacillus*^23–25^. Several probiotic *Lactobacillus* strains isolated from human intestinal tract, including *Lactobacillus johnsonii* and *Lactobacillus acidophilus*, have been well characterized with regard to their potential antimicrobial effects against major bacterial pathogens^26–28^. We observed a decrease of *Clostridium scindens*, which has been shown to inhibit the growth of *Clostridium difficile* through bile acid homeostasis and conversion of primary bile acids into secondary bile acids^29,30^. *Clostridium leptum* was previously reported to exhibit anti-inflammatory properties and a study reported that administration of a single strain of *Clostridium butyricum* resolved acute experimental colitis in mice through induction of IL-10, an anti-inflammatory cytokine^31,32^. *Eubacterium* (*E. rectale*, *E. ventriosum*, *E. plexicaudatum*) and *Ruminococcus* genera, which were both reported decreased in ulcerative colitis^33^, were also decreased one week after antibiotic and chemotherapy administration and restored by transplantation. We observed a depletion of *Butyricicoccus pullicaecorum* after antibiotic and chemotherapy administration. This depletion was still observed one week after chemotherapy discontinuation and restored after transplantation. *Butyricicoccus pullicaecorum* was recently reported to increase butyrate production and enhance intestinal epithelial barrier integrity in an *in vitro* model of Crohn’s disease suggesting an anti-inflammatory action^34^. In the same way, we observed a depletion of *Roseburia* producing bacteria (*R. faecis*, *R. intestinalis* and *R*. hominis) after antibiotics and chemotherapy. Such as *Butyricicoccus pullicaecorum* and *Fecalibacterium prausnitzii*, these species are supposed to reduce inflammation through butyrate production and are are reported dicreased in inflammatory bowel diseases^35–37^. A recent study also demonstrated, that an increase of *Barnesiella* was associated with an increase in short chain fatty acids and reduced inflammation in a rodent colitis-associated colorectal cancer model^38^. Conversely, we observed an overgrowth of pro-inflammatory species and well-recognized pathogens, as previously demonstrated in patients who received high dose of chemotherapy or patients hospitalized in intensive care units (*Escherichia coli*, *Staphylococcus aureus*, *Shigella*)^39^.

Microbiome functional profiles were critically altered by dual treatment with antibiotics and chemotherapy. Several functional modules were found to increase following antibiotic and chemotherapy administration in patients with inflammatory bowel disease, including cobalamin biosynthesis, riboflavin biosynthesis and modules linked to sugar transport^40^. Importantly, riboflavin is required for pH and oxidative stress homeostasis, through the biosynthesis of the reduced form of glutathione, an important antioxidant that alleviates the damage done by reactive oxygen species^37^. The direction of these changes suggests that the gut microbiota produced more glutathione following antibiotic treatment and chemotherapy administration to relieve the increased oxidative stress^41,42^.

As reported in Crohns disease, we found a significant increase of orthologs related to amino acid metabolism, bile acid metabolism and to fatty acid biosynthesis following antibiotic treatment and chemotherapy administration^43^. The increased bile acid biosynthesis was previously associated with intestinal inflammation and increased mucosal permeability^44,45^. We also observed an increase in modules involved in pathogenesis processes, such as secretion systems and adherence/invasion following antibiotic and chemotherapy treatment; these are known to be involved in the secretion of cell wall-degrading enzymes and the secretion of toxins^46^. We also observed, following antibiotic and chemotherapy administration, an increase in modules related to oxidative stress, known to be produced by Proteobacteria and enterococci^47^.

L-lactate dehydrogenase and arginine deiminase, which were decreased in mice that received antibiotics and chemotherapy, were previously reported to be associated with human health^48^. Furthermore, several pathways or enzymes related to short-chain fatty acids (SCFAs), which include acetate, propionate and butyrate, were decreased. SCFAs are the major source of energy for enterocytes, are involved in the maintenance of colonic mucosal health, and can resolve colitis. Loss of butyrate was previously associated with enteric infections, especially by *Clostridium difficile*^49^. A recent study also demonstrated that butyrate restoration through FMT improved intestinal epithelial cells, junctional integrity, decreased apoptosis, and mitigation of GVHD^50^. We also found a decrease in pathways and enzymes related to producing mucin, a key protein that composes the mucus layer. It is well known that mucus layer thickness and mucin production are qualitatively and quantitatively impaired during intestinal inflammation^51^. Several enzymes were associated with increased succinate metabolism, known to promote the expansion of *Clostridium difficile* and the development of colitis^52^.

Our results demonstrate that dual administration of antibiotics and chemotherapy lead to a critical decrease in key ‘health-promoting’ bacterial species^53^ and to an altered functional profile, increasing many previously described biomarkers of inflammation^54^. This disruption was stronger than when using chemotherapy alone. Although dual use prophylactic antibiotics and chemotherapy is standard practice in many hematopoietic stem cell or bone marrow transplant patients, our findings suggest that this practice compounds the dysbiosis caused by chemotherapy. However, mice that received FMT following antibiotic and chemotherapy administration did not exhibit taxonomic or functional dysbiosis one week after discontinuation of treatment. Importantly, in mice that received FMT, we did not find significant increase in taxonomic or functional pathways linked to intestinal inflammation or impaired mucosal barrier function. These findings suggest that FMT may be helpful in preventing acute intestinal inflammation and mucosal barrier dysfunction when receiving antibiotic and chemotherapy, such a treatment is frequently given to cancer patients. We believe that our results will inform customization and design of bacterial consortia for microbiota-targeted therapeutics in patients with cancer to prevent intestinal dysbiosis that can lead to life threatening complications such as BSI. The goal of future research will be to develop a custom probiotic cocktail of laboratory-grown microbes including the ‘health-promoting’ species identified here and elsewhere to reduce the risk of BSI in patients undergoing dual chemotherapy and antibiotic treatment.

## Materials and Methods

### Animals and housing

Seven week-old C57BL/6J mice were purchased from The Jackson Laboratory (Sacremento, CA), housed according to treatment group and cared for by Research Animal Resources (RAR) at the University of Minnesota, which is accredited by the American Association for Accreditation of Laboratory Animal Care. Mice were housed in individual cages and controlled environment with free access to food (standard chow containing 18% calories from fat and undetectable cholesterol) and water. All animal experiments were performed according to IACUC- and RAR-approved protocols (IBC Code Number 1501-32228 H).

### Study design and treatment administration

The aim of our study was to reproduce the patterns of cancer patients who exhibit intestinal dysbiosis induced by receiving courses of chemotherapy and antibiotics (Fig. 1).

#### Antibiotic treatment

Mice were treated with ampicillin (1 g/liter) in their drinking water during week 1, from days 1 to 7. A previous study reported that 7 days of ampicillin leads to a dysbiosis state^13^.

#### Chemotherapy

Mice were treated with one intraperitoneal injection of 5-Fluorouracil (5 FU) at 150 mg/kg, administered on day 8, one day after antibiotic discontinuation. A previous study showed that this dose causes dysbiosis and gastrointestinal mucositis and this drug was used in mouse models of bone marrow transplantation^55,56^.

#### Fecal transplantation

Feces from untreated mice were resuspended in phosphate-buffered saline (PBS) as previously described^13^. A 200 µl/day aliquot of the resuspended material (from an initial dilution of 5 g/ml feces) was given by oral gavage for 3 days, starting one day after chemotherapy administration, from days 9–11. The mice that did not receive FMT received oral gavages of water in order to match the stress of gavage manipulation during the same 3 days.

### Samples collection and DNA extraction

Fresh stools were collected in mice on Day 1, 8, 12 and 16 (Fig. 1). The samples were immediately frozen and stored at −80 °C until DNA extraction. Shallow shotgun sequencing of total stool DNA was done using Illumina the MiSeq platform at the University of Minnesota Genomic Center. Paired-end sequencing was performed using 2 × 250 base-pair reads following manufacturer recommendations. Libraries were prepared using Illumina barcodes (TruSeq DNA Sample Prep kit) and KAPA biosystems reagents (KAPA Library Preparation kit) as previously described^57^.

### Sequences analysis

Sequence reads were trimmed to the last base above a quality score of 20^58^. Taxonomic and functional predictions were performed using DIAMOND^59^ (version 0.9.18, default parameters -k 25 and -e 0.001). All reads were aligned against latest NCBI-nr database (downloaded in March 2018) using the DIAMOND-BLASTX algorithm. The significant matches were defined by e-value ≤ 10 * e-value of the top hits and were retained to define taxonomic groups^60,61^. Taxonomical levels were determined using the lowest common ancestor-based algorithm implemented in MEGAN^62^ (MEGAN Community Edition, version 6.10.10). A bit score of 86 was selected for filtering the results^63,64^. Functional profiling was performed using HUMAnN2 (version 0.11.1)^65^ and uniref50 database to establish KEGG orthologies and EC.

Alpha diversity analyses were performed using *vegan* R package (version 2.4-0). The Chao1 and observed-species biodiversity indices were computed and comparisons were performed using a nonparametric two-sample mean difference test with 999 Monte Carlo permutations. Beta diversity analyses were performed using Bray-Curtis distances in the *vegan* R package and were compared between groups with ANOSIM^50^. Relative abundances of species or functions were compared with non-parametric Mann Whitney and p-values were adjusted using false discovery rate. To identify the origin of the bacterial communities that composed the fecal microbiota in sample collected on Day 16 in mice, we used the Bayesian source tracking program SourceTracker (version 0.9.8) as described by Knights *et al*.^14^. This probabilistic OTU-based algorithm employs an iterative Bayesian approach to predict which OTUs in several potential source communities (here, samples from untreated mice, samples collected on Day 8 and Day 12) are likely to contribute to those in sink communities (here, samples collected on Day 16). SourceTracker was used to assess whether fecal samples collected one week after antibiotic and chemotherapy discontinuation in mice that did or did not receive FMT (sink communities) were mostly similar to microbiota from untreated mice, post antibiotic treatment microbiota or post FMT microbiota (source communities).

### Data accessibility

Raw data can be found in NCBI under bioproject id PRJNA389470.

## Electronic supplementary material

Supplementary material

Supplementary tables

## References

[1] van Vliet MJ, Harmsen HJM, de Bont ESJM, Tissing WJE (2010). The role of intestinal microbiota in the development and severity of chemotherapy-induced mucositis. PLoS Pathog..

[2] Marin M (2014). Bloodstream infections in neutropenic patients with cancer: differences between patients with haematological malignancies and solid tumours. J. Infect..

[3] Almyroudis NG (2005). Pre- and post-engraftment bloodstream infection rates and associated mortality in allogeneic hematopoietic stem cell transplant recipients. Transpl. Infect. Dis. Off. J. Transplant. Soc..

[4] Mikulska M (2012). Mortality after bloodstream infections in allogeneic haematopoietic stem cell transplant (HSCT) recipients. Infection.

[5] Blennow O, Ljungman P, Sparrelid E, Mattsson J, Remberger M (2014). Incidence, risk factors, and outcome of bloodstream infections during the pre-engraftment phase in 521 allogeneic hematopoietic stem cell transplantations. Transpl. Infect. Dis. Off. J. Transplant. Soc..

[6] Montassier E (2015). Chemotherapy-driven dysbiosis in the intestinal microbiome. Aliment. Pharmacol. Ther..

[7] Montassier E (2016). Pretreatment gut microbiome predicts chemotherapy-related bloodstream infection. Genome Med..

[8] Schelenz S, Nwaka D, Hunter PR (2013). Longitudinal surveillance of bacteraemia in haematology and oncology patients at a UK cancer centre and the impact of ciprofloxacin use on antimicrobial resistance. J. Antimicrob. Chemother..

[9] Ubeda C (2010). Vancomycin-resistant Enterococcus domination of intestinal microbiota is enabled by antibiotic treatment in mice and precedes bloodstream invasion in humans. J. Clin. Invest..

[10] Drekonja D (2015). Fecal Microbiota Transplantation for Clostridium difficile Infection: A Systematic Review. Ann. Intern. Med..

[11] Weingarden A (2015). Dynamic changes in short- and long-term bacterial composition following fecal microbiota transplantation for recurrent Clostridium difficile infection. Microbiome.

[12] Seekatz AM (2014). Recovery of the gut microbiome following fecal microbiota transplantation. mBio.

[13] Ubeda C (2013). Intestinal microbiota containing Barnesiella species cures vancomycin-resistant Enterococcus faecium colonization. Infect. Immun..

[14] Knights D (2011). Bayesian community-wide culture-independent microbial source tracking. Nat. Methods.

[15] Nagy-Szakal D (2012). Maternal micronutrients can modify colonic mucosal microbiota maturation in murine offspring. Gut Microbes.

[16] Zenewicz LA (2013). IL-22 deficiency alters colonic microbiota to be transmissible and colitogenic. J. Immunol. Baltim. Md 1950.

[17] Ward NL, Pieretti A, Dowd SE, Cox SB, Goldstein AM (2012). Intestinal aganglionosis is associated with early and sustained disruption of the colonic microbiome. Neurogastroenterol. Motil. Off. J. Eur. Gastrointest. Motil. Soc..

[18] Segata N (2012). Metagenomic microbial community profiling using unique clade-specific marker genes. Nat. Methods.

[19] Ounit R, Wanamaker S, Close TJ, Lonardi S (2015). CLARK: fast and accurate classification of metagenomic and genomic sequences using discriminative k-mers. BMC Genomics.

[20] Ounit R, Lonardi S (2016). Higher classification sensitivity of short metagenomic reads with CLARK-S. Bioinformatics.

[21] Tremlett H (2016). Gut microbiota in early pediatric multiple sclerosis: a case-control study. Eur. J. Neurol..

[22] Berg RD (1999). Bacterial translocation from the gastrointestinal tract. Adv. Exp. Med. Biol..

[23] Sokol H (2008). Faecalibacterium prausnitzii is an anti-inflammatory commensal bacterium identified by gut microbiota analysis of Crohn disease patients. Proc. Natl. Acad. Sci. USA.

[24] Patel B (2016). Lactobacillus acidophilus attenuates Aeromonas hydrophila induced cytotoxicity in catla thymus macrophages by modulating oxidative stress and inflammation. Mol. Immunol..

[25] Kalani M, Hodjati H, Sajedi Khanian M, Doroudchi M (2016). Lactobacillus acidophilus Increases the Anti-apoptotic Micro RNA-21 and Decreases the Pro-inflammatory Micro RNA-155 in the LPS-Treated Human Endothelial Cells. Probiotics Antimicrob. Proteins.

[26] Yamano T (2006). Improvement of the human intestinal flora by ingestion of the probiotic strain Lactobacillus johnsonii La1. Br. J. Nutr..

[27] Hsieh P-S (2012). Eradication of Helicobacter pylori infection by the probiotic strains Lactobacillus johnsonii MH-68 and L. salivarius ssp. salicinius AP-32. Helicobacter.

[28] Liévin-Le Moal V, Servin AL (2014). Anti-infective activities of lactobacillus strains in the human intestinal microbiota: from probiotics to gastrointestinal anti-infectious biotherapeutic agents. Clin. Microbiol. Rev..

[29] Greathouse KL, Harris CC, Bultman SJ (2015). Dysfunctional families: Clostridium scindens and secondary bile acids inhibit the growth of Clostridium difficile. Cell Metab..

[30] Buffie CG (2015). Precision microbiome reconstitution restores bile acid mediated resistance to Clostridium difficile. Nature.

[31] Kabeerdoss J, Sankaran V, Pugazhendhi S, Ramakrishna BS (2013). Clostridium leptum group bacteria abundance and diversity in the fecal microbiota of patients with inflammatory bowel disease: a case-control study in India. BMC Gastroenterol..

[32] Hayashi A (2013). A single strain of Clostridium butyricum induces intestinal IL-10-producing macrophages to suppress acute experimental colitis in mice. Cell Host Microbe.

[33] Ott SJ (2008). Dynamics of the Mucosa-Associated Flora in Ulcerative Colitis Patients during Remission and Clinical Relapse. J. Clin. Microbiol..

[34] Geirnaert, A. *et al*. Butyrate-producing bacteria supplemented *in vitro* to Crohn’s disease patient microbiota increased butyrate production and enhanced intestinal epithelial barrier integrity. *Sci. Rep*. **7** (2017). 10.1038/s41598-017-11734-8 PMC5597586 28904372

[35] Duncan SH, Hold GL, Barcenilla A, Stewart CS, Flint HJ (2002). Roseburia intestinalis sp. nov., a novel saccharolytic, butyrate-producing bacterium from human faeces. Int. J. Syst. Evol. Microbiol..

[36] Machiels K (2014). A decrease of the butyrate-producing species Roseburia hominis and Faecalibacterium prausnitzii defines dysbiosis in patients with ulcerative colitis. Gut.

[37] Morgan XC (2012). Dysfunction of the intestinal microbiome in inflammatory bowel disease and treatment. Genome Biol..

[38] Hu Y (2016). Manipulation of the gut microbiota using resistant starch is associated with protection against colitis-associated colorectal cancer in rats. Carcinogenesis.

[39] McDonald, D. *et al*. Extreme Dysbiosis of the Microbiome in Critical Illness. *mSphere* **1** (2016). 10.1128/mSphere.00199-16 PMC5007431 27602409

[40] Erickson AR (2012). Integrated metagenomics/metaproteomics reveals human host-microbiota signatures of Crohn’s disease. PloS One.

[41] Mulherin DM, Thurnham DI, Situnayake RD (1996). Glutathione reductase activity, riboflavin status, and disease activity in rheumatoid arthritis. Ann. Rheum. Dis..

[42] Yassour M (2016). Sub-clinical detection of gut microbial biomarkers of obesity and type 2 diabetes. Genome Med..

[43] Jansson J (2009). Metabolomics reveals metabolic biomarkers of Crohn’s disease. PloS One.

[44] Lowes S, Simmons NL (2001). Human intestinal cell monolayers are preferentially sensitive to disruption of barrier function from basolateral exposure to cholic acid: correlation with membrane transport and transepithelial secretion. Pflugers Arch..

[45] Chen F (2002). Inflammatory-mediated repression of the rat ileal sodium-dependent bile acid transporter by c-fos nuclear translocation. Gastroenterology.

[46] Jha G, Rajeshwari R, Sonti RV (2005). Bacterial type two secretion system secreted proteins: double-edged swords for plant pathogens. *Mol. Plant-Microbe Interact*. MPMI.

[47] Sherrill C, Fahey RC (1998). Import and metabolism of glutathione by Streptococcus mutans. J. Bacteriol..

[48] Belda-Ferre P (2015). The human oral metaproteome reveals potential biomarkers for caries disease. Proteomics.

[49] Furusawa Y (2013). Commensal microbe-derived butyrate induces the differentiation of colonic regulatory T cells. Nature.

[50] Mathewson ND (2016). Gut microbiome-derived metabolites modulate intestinal epithelial cell damage and mitigate graft-versus-host disease. Nat. Immunol..

[51] Corfield AP (2000). Mucins and mucosal protection in the gastrointestinal tract: new prospects for mucins in the pathology of gastrointestinal disease. Gut.

[52] Ferreyra JA (2014). Gut microbiota-produced succinate promotes C. difficile infection after antibiotic treatment or motility disturbance. Cell Host Microbe.

[53] Lloyd-Price J, Abu-Ali G, Huttenhower C (2016). The healthy human microbiome. Genome Med..

[54] Gilbert JA (2016). Microbiome-wide association studies link dynamic microbial consortia to disease. Nature.

[55] Noach EJK (2003). Chemotherapy prior to autologous bone marrow transplantation impairs long-term engraftment in mice. Exp. Hematol..

[56] Goebel WS (2004). A murine model of antimetabolite-based, submyeloablative conditioning for bone marrow transplantation: biologic insights and potential applications. Exp. Hematol..

[57] Clayton JB (2016). Captivity humanizes the primate microbiome. Proc. Natl. Acad. Sci. USA.

[58] Quast C (2013). The SILVA ribosomal RNA gene database project: improved data processing and web-based tools. Nucleic Acids Res..

[59] Buchfink B, Xie C, Huson DH (2015). Fast and sensitive protein alignment using DIAMOND. Nat. Methods.

[60] Qin J (2010). A human gut microbial gene catalogue established by metagenomic sequencing. Nature.

[61] Cui, X. *et al*. Metagenomic and metabolomic analyses unveil dysbiosis of gut microbiota in chronic heart failure patients. *Sci. Rep*. **8** (2018). 10.1038/s41598-017-18756-2 PMC5766622 29330424

[62] Huson DH, Auch AF, Qi J, Schuster SC (2007). MEGAN analysis of metagenomic data. Genome Res..

[63] Urich T (2008). Simultaneous Assessment of Soil Microbial Community Structure and Function through Analysis of the Meta-Transcriptome. PLOS ONE.

[64] Rea MC (2012). Clostridium difficile Carriage in Elderly Subjects and Associated Changes in the Intestinal Microbiota. J. Clin. Microbiol..

[65] Abubucker S (2012). Metabolic reconstruction for metagenomic data and its application to the human microbiome. PLoS Comput. Biol..

