# Supplementary material for: Fecal microbiota transplantation reverses antibiotic and chemotherapy-induced gut dysbiosis in mice
Source: Sci Rep. 2018 Apr 18;8:6219. doi: 10.1038/s41598-018-24342-x (PMC5906603; doi:10.1038/s41598-018-24342-x)
Supplement: Supplementary file 1 — Supplementary material [file 41598_2018_24342_MOESM1_ESM.doc]

Supplementary Materials for

# **Fecal microbiota transplantation reverses antibiotic and chemotherapy-induced gut dysbiosis in mice**

Quentin Le Bastard^1^, Tonya Ward^2^, Dimitri Sidiropoulos^2^, Benjamin M. Hillmann^3^, Chan Lan Chun^2,4^, Michael J. Sadowsky^2,5^, Dan Knights^2,3*^, and Emmanuel Montassier^1*^

**This PDF file includes:**

Supplementary discussion, Figs. S1 to S3, Captions for Additional Data tables S1 to S13 (Excel file)

**Supplementary discussion**

***Decrease of Lactobacillus strains following antibiotic and chemotherapy.*** Several probiotic *Lactobacillus* strains isolated from human intestinal microbiota, including *Lactobacillus johnsonii* and *Lactobacillus acidophilus,* have been well characterized with regard to their potential antimicrobial effects against the major gastric and enteric bacterial pathogens [1–3]. In a prospective randomized trial, concomitant therapy of *Lactobacillus acidophilus* with amoxicillin/clavulanate was associated with a significant decrease in patient complaints of gastrointestinal side effects and yeast superinfection [4].

***Decrease of Clostridium scidens following antibiotic and chemotherapy.*** Furthermore, we observed a decrease of *Clostridium scindens,* which has been shown to inhibit the growth of *Clostridium difficile* through bile acid homeostasis and conversion of primary bile acids into secondary bile acids [5,6]. Indeed, *C. scindens* expresses enzymes which are crucial for secondary bile acid synthesis, enzymes that are unfrequently carried by other bacteria [7,8]. Here, we confirmed this finding as we showed that secondary bile acid biosynthesis (ko00121) was decreased immediately after antibiotic treatment and still depleted one week after antibiotic and chemotherapy discontinuation in mice that did not receive FMT. *C. scindens* was also reported to exhibit anti-inflammatory properties through the induction of anti-inflammatory Treg expansion [9].

***Increase of Clostridium strains and Barnesiella following FMT.*** In mice that received FMT, we found an increase in *Clostridium leptum* and *Clostridium butyricum* immediately after FMT. *Clostridium leptum* was previously reported to exhibit anti-inflammatory properties and a study reported that administration of a single strain of *Clostridium butyricum* resolved acute experimental colitis in mice through induction of IL-10, an anti-inflammatory cytokine [10,11].

It was previously reported that Barnesiellaceae-enriched fecal microbiota is protective against BSI in cancer patients and that *Barnesiella* genus is associated with protection against vancomycin-resistant *Enterococcus* domination in the gut [12,13]. A recent study also demonstrated, in a rodent colitis-associated colorectal cancer, that the increase of *Barnesiella* was associated with increased in short chain fatty acids and reduced inflammation [14].

***Increase of functional modules associated with inflammation.*** We found that community function is critically altered by tandem antibiotics and chemotherapy. Several modules found increased following antibiotic and chemotherapy administration were reported increased in patients with inflammatory bowel disease, including cobalamin biosynthesis, riboflavin biosynthesis and modules linked to sugar transport [15].

Importantly, riboflavin is required for pH and oxidative stress homeostasis, through the biosynthesis of the reduced form of glutathione, an important antioxidant that alleviates the damage done by reactive oxygen species [16].

Here, we also observed that glutathione biosynthesis was increased in mice submitted to antibiotic and chemotherapy treatment. The direction of these changes suggests that the gut microbiota produced more glutathione following antibiotic treatment and chemotherapy administration to relieve the increased oxidative stress [17,18].

We also found, as reported in Crohn disease, a significant increase following antibiotic treatment and chemotherapy administration of modules related to amino acid metabolism (Tyrosine biosynthesis, Tryptophan biosynthesis, Phenylalanine biosynthesis), bile acid metabolism (Conjugated bile acid biosynthesis choloyl CoA taurocholate glycocholate) and to fatty acid biosynthesis (Fatty acid biosynthesis initiation, Fatty acid biosynthesis elongation) [19]. A study reported a correlation between amino acid levels and abundance of mucosal *E. coli* in the gut in Crohn disease patients, which is one of the microorganisms we also found increased following antibiotic or chemotherapy treatment. The increased bile acid biosynthesis was previously associated with intestinal inflammation and increased mucosal permeability [20,21]. On the same hand, increase of fatty acid biosynthesis was known to mediate inflammation and to be increased in experimental colitis [22].

We also observed an increase in modules involved in pathogenesis processes, such as secretion systems and adherence/invasion (Type II general secretion system, Type III secretion system, Type VI secretion system, EHEC pathogenicity signature Shiga toxin) following antibiotic and chemotherapy treatment, known to be involved in the secretion of cell wall-degrading enzymes and the secretion of toxins [23]. These functions were related to adherent-invasive *E. coli*, which have been also observed to be increased in Crohn disease [16,24]. Therefore, following antibiotic treatment and chemotherapy, the functional dysbiosis lead to a functional profile with pathobiont-like properties, as previously reported in intestinal inflammation [16]. We also observed, after antibiotic and chemotherapy administration, an increase in modules related to oxidative stress (Glutathione transport system, Sulfate transport system), known to be produced by Proteobacteria and enterococci [25]. Previous studies reported that redox stress confer competitive advantage to *Salmonella* and enterohemorrhagic *E. coli* [26,27].

L-lactate dehydrogenase and arginine deiminase, decreased in mice that received antibiotic and chemotherapy, were previously reported to be associated with human health [28].

Furthermore, several decreased pathways or Enzyme Commission were related to short-chain fatty acids (SCFAs) which include acetate, propionate and butyrate (Propanoate metabolism, Butanoate metabolism, Butyrate CoA ligase, Pyruvate kinase, Butyrate kinase, Acetate CoA-transferase). SCFAs are the major source of energy for enterocytes, involved in the maintenance of colonic mucosal health and can resolve colitis. Loss of butyrate was previously associated with enteric infections, especially *Clostridium difficile* infection [9]. A recent study also demonstrated that butyrate restoration through FMT improved intestinal epithelial cells, junctional integrity, decreased apoptosis, and mitigated GVHD [29]. We also found a decrease in pathways or Enzyme Commission related to mucin production, key proteins that compose the mucus layer (GDP-L-fucose synthase, GDP-4-dehydro-6-deoxy-D-mannose reductase, Glucosamine-6-phosphate deaminase). It is well known that mucus layer and mucin production are qualitatively and quantitatively impaired during intestinal inflammation [30]. Several EC were associated with increased succinate metabolism, known to promote the expansion of *Clostridium difficile* and the development of colitis [31].

**Supplementary** **Figure S1. Alpha and beta diversity are not significantly modified over time in untreated mice. (A)** Comparison of alpha diversity indexes for each collection time points in untreated mice. Chao1 index (left) and number of unique observed species (right). **(B)** Species beta diversity comparison of the gut microbiomes of fecal samples collected at different time points in untreated mice. Principal coordinate analysis (PCoA) of Bray Curtis distances. Proportion of variance explained by each principal coordinate axis is denoted in the corresponding axis label.

**S1A**

NS


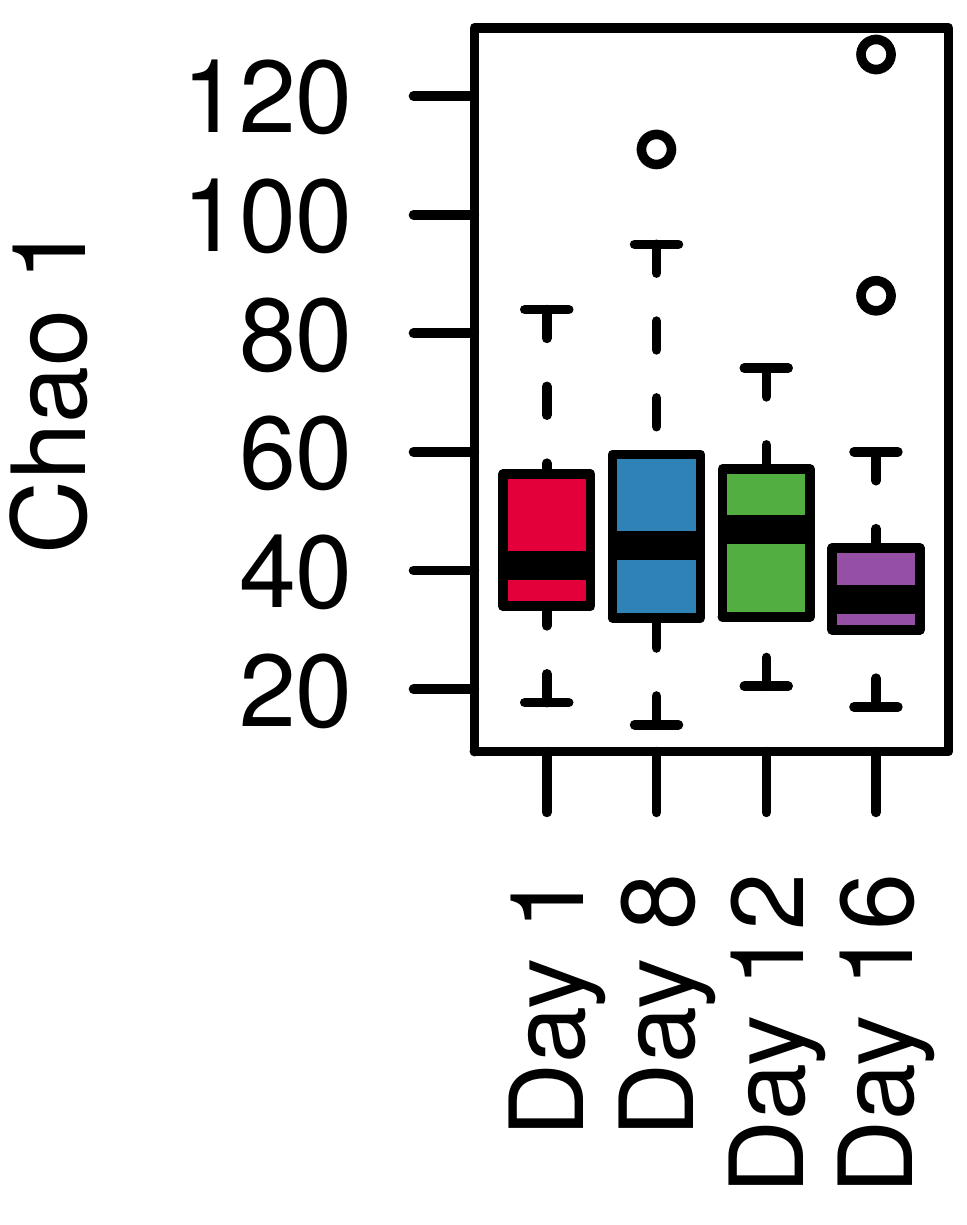

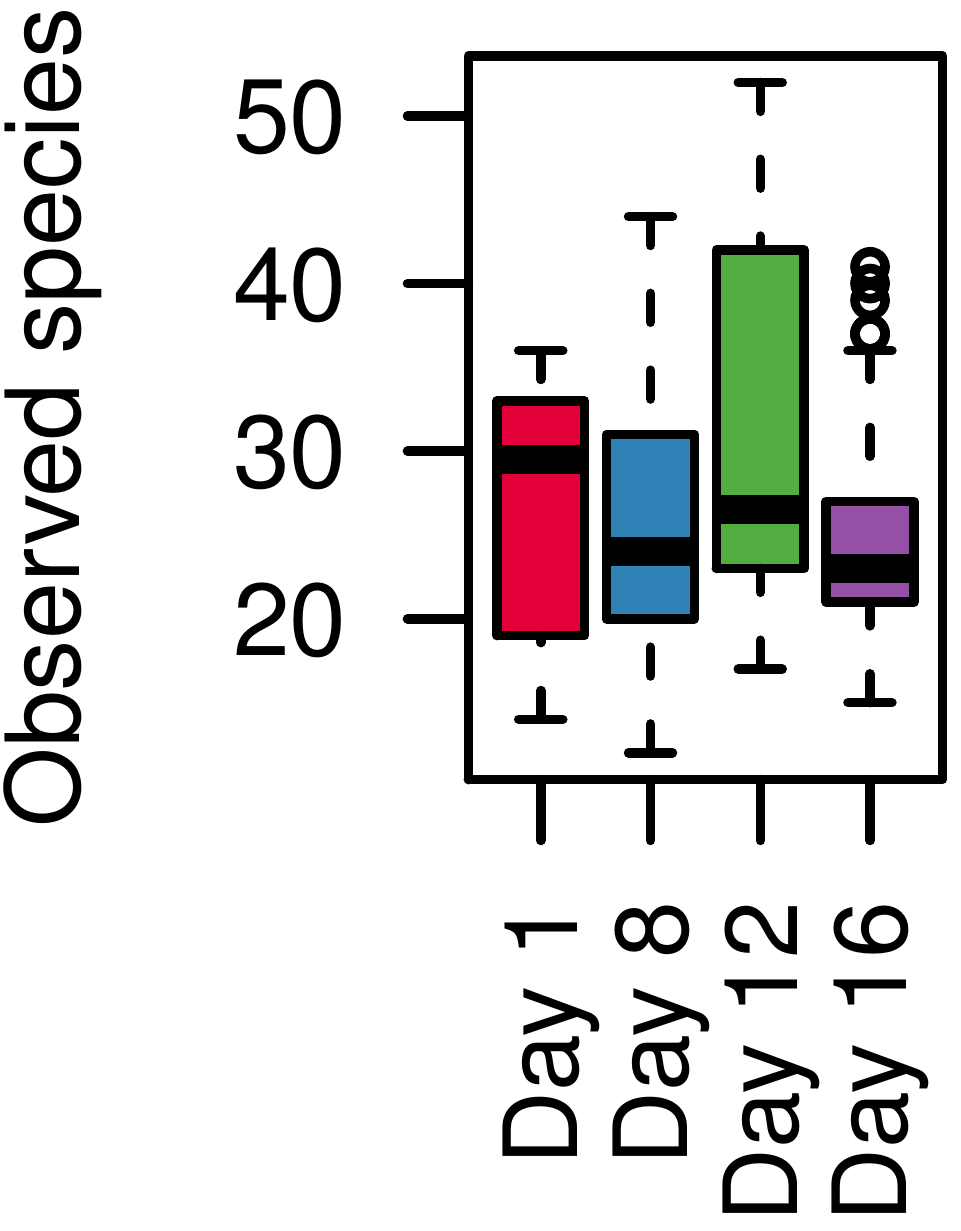


NS

**S1B**


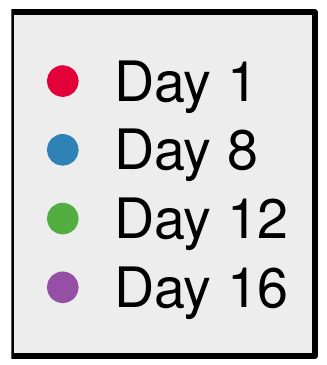


**Untreated mice**

**Supplementary** **Figure S2.** Chemotherapy alone also disrupts microbiota architecture. Species beta diversity comparison of the gut microbiomes of fecal samples collected in mice exposed to chemotherapy on Day 8. Principal coordinate analysis (PCoA) of Bray Curtis distances. Proportion of variance explained by each principal coordinate axis is denoted in the corresponding axis label.

**Chemotherapy**

**Chemotherapy and FMT**


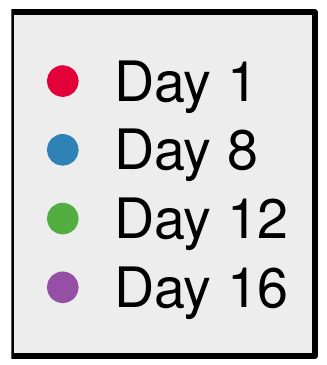

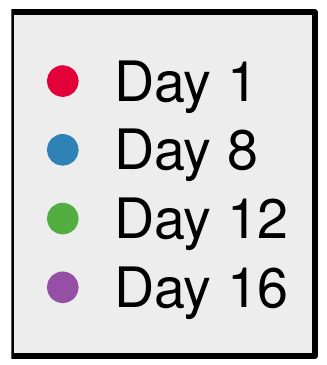


**S2**

**Supplementary** **Figure S3. Taxonomic profiling using MetaPhlAn2 reports the same genii distribution as DIAMOND profiling with a clear predominance of Lactobacilli.** Each plot shows, for one mouse, the longitudinal follow up of the 10 most abundant bacterial genera. The four plots on top represent mice exposed to antibiotic treatment from days 1 to 7 and chemotherapy on day 8. The four bottom plots represent mice that received the same regimen and FMT from days 9 to 11.

**S3**

**Table S1.** Taxonomic changes at genus level between untreated mice and mice that receive antibiotic treatment for one week.

**Table S2.** Taxonomic changes at species level between untreated mice and mice that receive antibiotic treatment for one week.

**Table S3.** Taxonomic changes at species level between untreated mice and mice that receive antibiotic treatment for one week and one intraperitoneal injection of 5FU.

**Table S4.** Taxonomic changes at species level between untreated mice and mice that receive antibiotic treatment for one week and one intraperitoneal injection of 5FU, one week after discontinuation of antibiotics and chemotherapy.

**Table S5.** Taxonomic changes at species level between untreated mice and mice that receive antibiotic treatment for one week, one intraperitoneal injection of 5FU and fecal microbiota transplantation.

**Table S6.** KEGG orthologs that changed between untreated mice and mice that receive antibiotic treatment for one week.

**Table S7.** KEGG orthologs that changed between untreated mice and mice that receive antibiotic treatment for one week and one intraperitoneal injection of 5FU.

**Table S8.** KEGG orthologs that changed between untreated mice and mice that receive antibiotic treatment for one week and one intraperitoneal injection of 5FU, one week after discontinuation of antibiotics and chemotherapy.

**Table S9.** Enzyme Commission that changed between untreated mice and mice that receive antibiotic treatment for one week.

**Table S10.** Enzyme Commission that changed between untreated mice and mice that receive antibiotic treatment for one week and one intraperitoneal injection of 5FU.

**Table S11.** Enzyme Commission that changed between untreated mice and mice that receive antibiotic treatment for one week and one intraperitoneal injection of 5FU, one week after discontinuation of antibiotics and chemotherapy.

**Table S12.** KEGG modules that changed between untreated mice and mice that receive one intraperitoneal injection of 5FU alone.

**Table S13.** Enzyme Commission that changed between untreated mice and mice that receive one intraperitoneal injection of 5FU alone.
